# Complete chloroplast genome of a rare and endangered plant species *Osteomeles subrotunda*: genomic features and phylogenetic relationships with other Rosaceae plants

**DOI:** 10.1080/23802359.2021.1881835

**Published:** 2021-03-11

**Authors:** Ming Jiang, Junfeng Wang, Minghui Chen, Huijuan Zhang

**Affiliations:** a College of Life Sciences, Taizhou University, Taizhou, P. R. China; b Scientific Research Management Center, East China Medicinal Botanical Garden, Lishui, P. R. China

**Keywords:** *Osteomeles subrotunda*, rare and Endangered species, chloroplast genome, phylogenetic analysis

## Abstract

*Osteomeles subrotunda* is a rare and endangered plant species with extremely small populations. In our study, we sequenced the complete chloroplast (CP) genome of *O. subrotunda* and described its structural organization, and performed comparative genomic analyses with other Rosaceae CP genomes. The plastome of *O. subrotunda* was 159,902 bp in length with 36.6% GC content and contained a pair of inverted repeats of 26,367 bp which separated a large single-copy region of 87,933 bp and a small single-copy region of 19,235 bp. The CP genome included 130 genes, of which 85 were protein-coding genes, 37 were transfer RNAs, and eight were ribosomal RNAs. Two genes, *rps19* and *ycf1*, which are located at the borders of IRB/SSC and IRB/LSC, were presumed to be pseudogenes. A total of 61 SSRs were detected, of which, 59 loci were mono-nucleotide repeats, and two were di-nucleotide repeats. The phylogenic analysis indicated that the 14 Rosaceae species were divided into three groups, among which *O. subrotunda* grouped with *P. rupicola*, *E. japonica*, *P. pashia*, *C. japonica*, *S. torminalis*, and *M. florentina*, and it was found to be a sister clade to *C. japonica*. Our newly sequenced CP genome of *O. subrotunda* will provide essential data for further studies on population genetics and biodiversity.

## Introduction

Rosaceae, the rose family, composed of over 100 genera and 3000 species, is a medium-sized flowering plant family with significant economic and scientific importance (Jung and Main 2014). Rosaceae family contains a considerable number of fruit plants, ornamentals, and herbs, which greatly benefit people all over the world (Wu et al. 2003). *Osteomeles* is a small genus in the rose family, comprising only about five species, including *O. subrotunda*, *O. anthyllidifolia*, and *O. schwerinae*, which are native to eastern Asia and Polynesia (Hsieh and Chaw 1996). There are very few reports regarding *Osteomeles* plants, and most of them focus on *O. schwerinae*, a traditional medicinal plant widely used in Asia countries. Lee et al. (2010) isolated two flavonol glucosides, hyperoside and quercitrin, from *O. schwerinae* by using semi-preparative high-speed counter-current chromatography separation technique. Sohn et al. (2018) reported that *O. schwerinae* extract inhibited the accumulation of extracellular matrix and the proliferation of rat glomerular mesangial cells. *O. subrotunda* distributes only in Ryukyu Islands in Japan and Renhua in China (Wu et al. 2003). In recent years, new distributions were found in several islands in Zhejiang Province, China, and according to our long-term investigation, the total plant number was found to be less than 300. *O. subrotunda* was now listed as a plant species with extremely small populations as well as a key protected wild plant in Zhejiang Province.

Chloroplasts (CPs) are essential organelles that carry out photosynthesis to produce sugars and oxygen in eukaryotic plant cells; Moreover, they synthesize fatty acids, terpenes, and amino acids for multiple functions (Waters and Langdale 2009; Dorrell and Howe 2012; Shi and Theg 2013). Like a mitochondrion, a CP possesses its unique genome, which is organized into a single circular chromosome (Martin et al. 2013; Allen 2015). As compared to nuclear genomes, the CP genomes of land plants are more conserved in structural organization, containing a large single-copy (LSC) region, a small single-copy (SSC) region, and two inverted repeat (IR) regions (Daniell et al. 2016). In higher plants, the size of CP genomes varies from 70 to 220 kb, which contains 110–130 genes like transfer RNAs (tRNA), ribosomal RNAs (rRNA), and protein-coding genes (Whittall et al. 2010; Dong et al. 2013).

In recent years, the complete CP genomes of several Rosaceae plants were sequenced and characterized, and these included *Rosa praelucens* (Jian, Zhang, Zhang, et al. 2018), *Fragaria*×*ananassa* (Cheng et al. 2017), and *Hagenia abyssinica* (Gichira et al. 2017). However, the CP genome sequence of *Osteomeles* remained uncharacterized. In our current study, we report the complete CP genome sequences of *O. subrotunda*, an endangered Rosaceae plant in China, by analyzing CP genome characteristics and performing comparative analysis against other 13 Rosaceae species. The completely sequenced CP genome of *O. subrotunda* will provide a valuable source for population genetics and biodiversity studies in the future.

## Materials and methods

### Plant material and DNA extraction

Fresh leaves were collected from Toumen Island (28°41.132′N, 121°46.502′E), Zhejiang Province, China. A voucher specimen coded CHS2017108 was deposited at the Molecular Biology Laboratory in Taizhou University. Approximately, 0.5 g of leaves were ground into a fine powder using liquid nitrogen in a sterile pestle and mortar, and genomic DNA was extracted following the cetyltrimethylammonium bromide (CTAB) protocol (Doyle and Doyle 1987).

### DNA sequencing and sequence assembly

A DNA library was prepared and was then sequenced using an Illumina Hiseq X Ten system (Illumina, San Diego, CA, USA). Approximately, 3.6 Gb raw data of 150 bp paired-end reads were obtained, and they were then filtered by NGS QC Toolkit v2.3.3 to obtain clean reads (Patel and Jain 2012). NOVOPlasty was applied to assemble the plastome (Dierckxsens et al. 2017).

### Chloroplast genome annotation

Annotation of the complete CP genome was performed with the CPGAVAS2 (http://47.96.249.172:16019/analyzer/annotate) (Shi et al. 2019), and the borders of each gene were manually adjusted. The circular CP genome map was obtained using an online tool namely OrganellarGenomeDraw (OGDRAW, http://ogdraw.mpimp-golm.mpg.de) (Lohse et al. 2013).

### Simple sequence repeat analysis

MIcroSAtellite Identification Tool (MISA) was applied to identify simple sequence repeats (SSRs) in *O. subrotunda* complete CP genome (Thiel et al. 2003). To perform SSR detection, 1–6 bp nucleotide motifs were considered, and the minimum repeat unit size was defined as follows: 10 for mono-nucleotides, six for di-nucleotides, and five for tri-, tetra-, penta-, and hexa-nucleotides.

### Phylogenetic analysis

The complete CP genome sequence of *Glycine falcata* (NC_021649.1) was retrieved from NCBI and served as an outgroup. The genome sequences were aligned by performing a multiple sequence alignment using MAFFT v7.388 (Katoh and Standley 2013). The best-fit DNA substitution model for maximum-likelihood (ML) analysis was selected by running program jModelTest 2.1.9 under the Akaike information criterion (AIC) (Darriba et al. 2012). An ML tree was constructed by using program PhyML 3.1 using the best-fit model, GTR + G+I, with 1000 bootstrap replicates (Guindon et al. 2010).

## Results

### Genome sequencing and assembly

**Figure 1.**
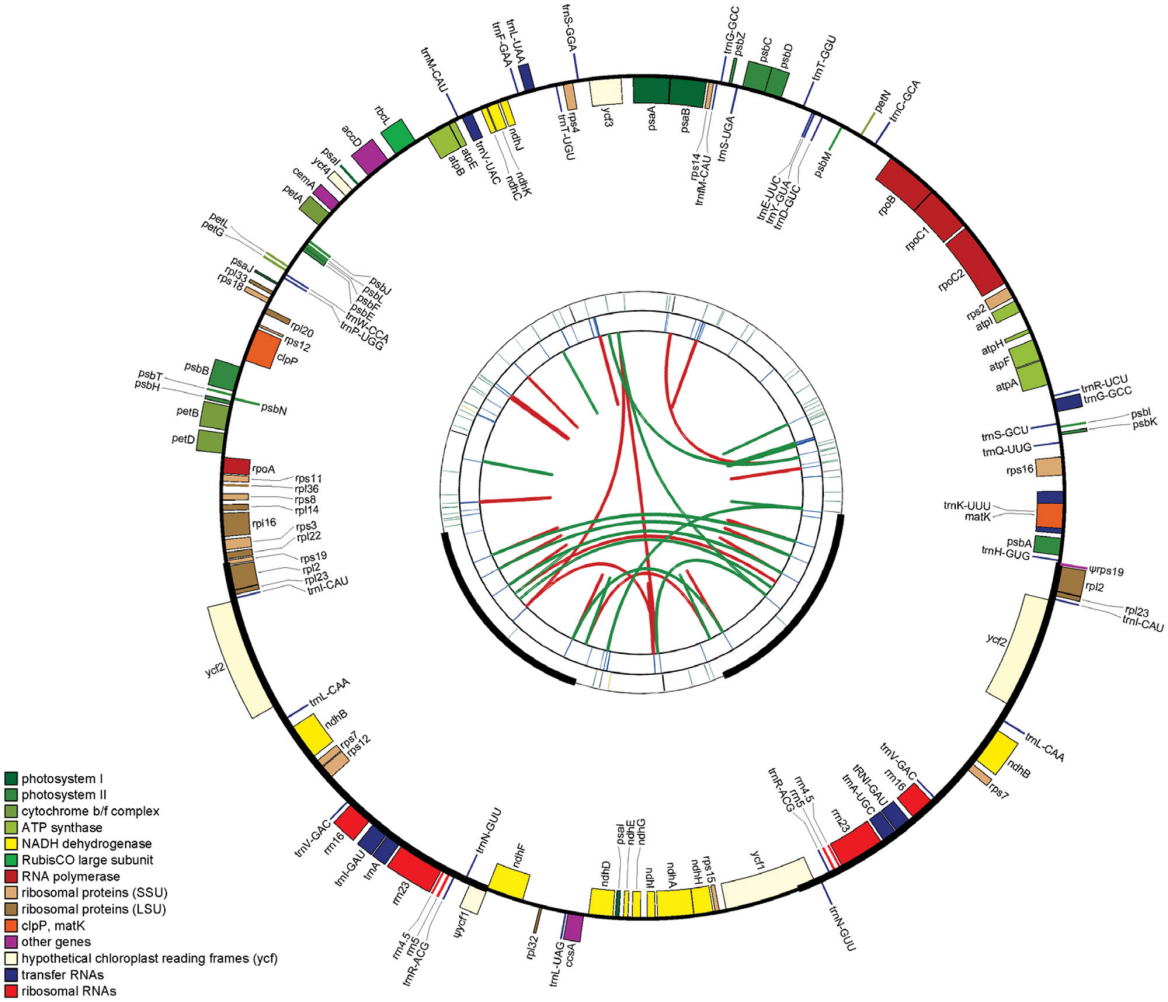
The chloroplast genome of *Osteomeles subrotunda*. From the center going outward, the four circles indicate scattered forward and reverse repeats, tandem repeats, microsatellite sequences identified, and gene structure of the plastome.

Overall, we yielded 11,924,123 clean reads (150 bp in average length) by removing unqualified reads in raw reads, and after de novo assembly, a circular contig was assembled with Novoplasty. The complete CP genome of *O. subrotunda* was 159,902 bp in length, which exhibited a typical quadripartite structure, including an LSC region (87,933 bp), an SSC region (19,235 bp), and two copies of IRs (26,367 bp). The GC content of *O. subrotunda* CP genome was 36.6%, which was unevenly distributed across the four regions; however, GC contents of intergenic regions and introns were lower. The highest GC content was observed in IRs (42.7%), followed by the LSC region (34.3%) and the SSC region (30.4%) (Figure 1).

### Genome features of *Osteomeles subrotunda*

The complete CP genome of *O. subrotunda* consisted of 130 genes, of which 85 were protein-coding genes, 37 were tRNA genes, and eight were rRNA genes (Table S1). Based on gene functions, these genes could be divided into four categories, genes related to self-replication, genes related to photosynthesis, genes related to biosynthesis of amino acids, fatty acids, and carboxylates, and several functionally unknown genes (*ycf1*, *ycf2*, and *ycf4*). Among these genes, five protein-coding genes (*rpl2*, *rpl23*, *rps7*, *ndhB*, and *ycf2*), eight tRNAs (*trnA-UGC*, *trnG-GCC*, *trnI-CAU*, *trnI-GAU*, *trnL-CAA*, *trnN-GUU*, *trnR-ACG*, and *trnV-GAC*), and four rRNAs (*rrn4.5*, *rrn5*, *rrn16*, and *rrn23*) included two copies. There were 11 protein-coding genes and nine tRNA genes harbored only one intron, while three protein-coding genes (*rps12*, *clpP*, and *ycf3*) contained two introns (Table 1). Two genes, *rps19* and *ycf1*, which are located at the borders of IRB/SSC and IRB/LSC, were presumed to be pseudogenes. The *rps12* gene in *O. subrotunda* consisted of three exons, and the intron between exons 1 and 2 was trans-splicing.

**Table 1.** List of genes in the chloroplast genome of *Osteomeles subrotunda*.

Group of genes	Name of genes	Total number
Large subunit of ribosomal proteins	*rpl2* (×2)^a^, *rpl14*, *rpl16*^a^, *rpl20*, *rpl22*, *rpl23* (×2), *rpl32*, *rpl33*, *rpl36*	11
Small subunit of ribosomal proteins	*rps2*, *rps3*, *rps4*, *rps7* (×2), *rps8*, *rps11*, *rps12*^b^, *rps14*, *rps15*, *rps16*^a^, *rps18*, *rps19*, ψ*rps19*	14
DNA-dependent RNA polymerase	*rpoA*, *rpoB*, *rpoC1*^a^, *rpoC2*	4
Ribosomal RNA genes	*rrn4.5* (×2), *rrn5* (×2), *rrn16* (×2), *rrn23* (×2)	8
Transfer RNA genes	*trnA-UGC* (×2)^a^, *trnC-GCA*, *trnD-GUC*, *trnE-UUC*, *trnF-GAA*, *trnfM-CAU*, *trnG-GCC* (×2)^a^, *trnH-GUG*, *trnI-CAU* (×2), *trnI-GAU* (×2)^a^, *trnK-UUU*^a^, *trnL-CAA* (×2), *trnL-UAA*^a^, *trnL-UAG*, *trnM-CAU*, *trnN-GUU* (×2), *trnP-UGG*, *trnQ-UUG*, *trnR-ACG* (×2), *trnR-UCU*, *trnS-GCU*, *trnS-GGA*, *trnS-UGA*, *trnT-GGU*, *trnT-UGU*, *trnV-GAC* (×2), *trnV-UAC*^a^, *trnW-CCA*, *trnY-GUA*	37
Photosystem I	*psaA*, *psaB*, *psaC*, *psaI*, *psaJ, ycf3*^b^	6
Photosystem II	*psbA*, *psbB*, *psbC*, *psbD*, *psbE*, *psbF*, *psbH*, *psbJ*, *psbK*, *psbL*, *psbI*, *psbM*, *psbN*, *psbT*, *psbZ*	15
Cytochrome b6/f complex	*petA*, *petB*^a^, *petD*^a^, *petG*, *petL*, *petN*	6
NADH dehydrogenase	*ndhA*^a^, *ndhB (×2)*^a^, *ndhC*, *ndhD*, *ndhE*, *ndhF*, *ndhG*, *ndhH*, *ndhI*, *ndhJ*, *ndhK*	12
ATP synthase	*atpA*, *atpB*, *atpE*, *atpF*^a^, *atpH*, *atpI*	6
Rubisco	*rbcL*	1
Translational initiation factor	*infA*	1
Maturase	*matK*	1
Protease subunit P	*clpP*^b^	1
Envelop membrane protein	*cemA*	1
Subunit acetyl-CoA carboxylate	*accD*	1
c-type cytochrome synthesis gene	*ccsA*	1
Conserved open reading frames	*ycf1*, ψ*ycf1*, *ycf2 (×2)*, *ycf4*	5
Total		130

(ψ) pseudogene.

^a^ One intron.

^b^ Two introns.

### IR expansion and contraction

**Figure 2.**
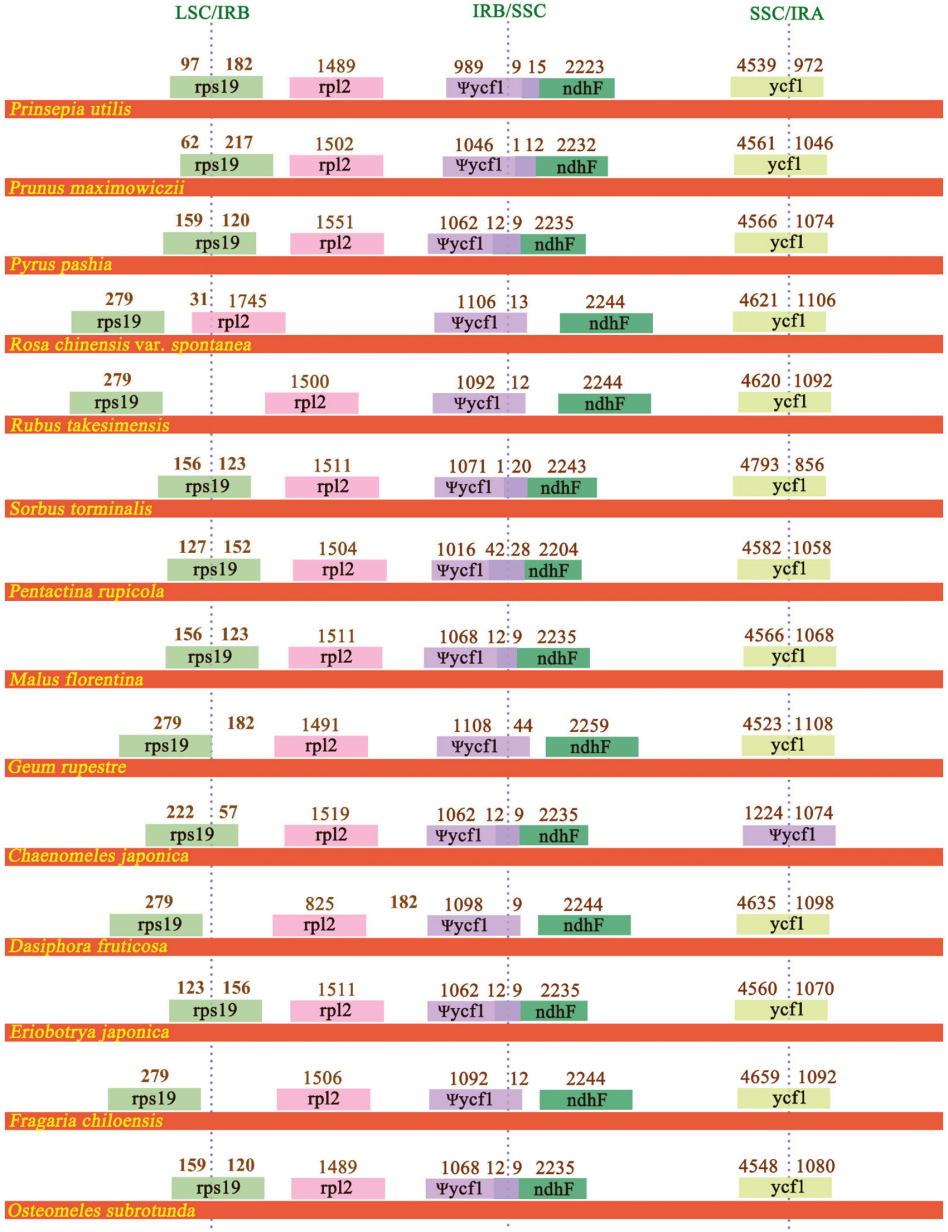
Structural variations at inverted-repeat and single-copy borders in 14 Rosaceae chloroplast genomes. The figure features are not to scale regarding sequence length.

As compared to 13 Rosaceae CP genome sequences, the length of IR in *O. subrotunda* was shorter that these of *P. maximowiczii* (26,436 bp), *S. torminalis* (26,416 bp), *P. pashia* (26,386 bp), and *M. florentina* (26,381 bp), but longer than other CP genomes (25,311–26,326 bp) (Table S1). Structural variations were observed in LSC/IR/SSC boundaries. In *O. subrotunda* CP genome, the IRA/SSC and IRB/SSC borders were present within upstream regions of the two *ycf1* genes, and the *ycf1* which IRA/SSC border located was a complete gene with a length of 5628 bp, while the other *ycf1* in which IRA/SSC border located was 1089 bp in length. The same phenomena were found with the two *rps19* genes. The LSC/IRB border located in the complete coding region of the *rps19* gene which was 279 bp in length, while the ycf1 where LSC/IRA border located was proved to be a pseudogene with its 3′ region truncated (Figure 2).

Expansions or contractions of IR regions were observed among the 14 Rosaceae CP genome sequences. For all species, the IRB/SSC border was in the 3′ region of the *ycf1*, and created an *ycf1* pseudogene with a length of 1013 (*P. utilis*) to 1152 bp (*G. rupestre*). For *P. rupicola*, *P. utilis*, *P. maximowiczii*, *P. pashia*, *C. japonica*, *E. japonica*, *M. florentina*, *S. torminalis*, and *O. subrotunda* CP genomes, the LSC/IRB border located within the coding sequence of *rps19*, while for *D. fruticose*, *F. chiloensis*, *G. rupestre*, and *R. takesimensis*, the LSC/IRB border was seen to locate in the intergenic region of *rps19* and *rpl2*. In *R. chinensis* var. *spontanea* CP genome sequence, the LSC/IRB border located within *rpl12*. SSC/IRAs located within the *ycf1* genes except for the *C. japonica* CP genome, in which a 3′-truncated *ycf1* pseudogene with a length of 2298 bp was produced by the border (Figure 2). The *ndhF* genes of *P. rupicola*, *P. utilis*, *P. maximowiczii*, *P. pashia*, *C. japonica*, *E. japonica*, *M. florentina*, and *S. torminalis*, were extended and overlapped with pseudogene *ycf1*.

### SSR analysis

A total of 61 SSRs were detected in the plastid genome of *O. subrotunda*. Of these, 59 loci were mono-nucleotide repeats, and two were di-nucleotide repeats (Table S2). Among 59 mono-nucleotides, 26A stretches, one C stretch, and 32T stretches were identified; however, no G stretch was found. The two di-nucleotides were both AT stretches, including a six repeat motif and a seven repeat motif, respectively. The length of the SSRs ranged from 10 to 20 bp, and most of them located in intergenic or intron regions. Seven genes, *ycf1*, *atpH*, *trnI-GAU*, *trnG-GCC*, *rpoC2*, *rpoB*, and *atpB*, contained one or two SSRs. Only two SSRs were observed harboring in IRA and IRB, respectively.

### Phylogenetic analysis

**Figure 3.**
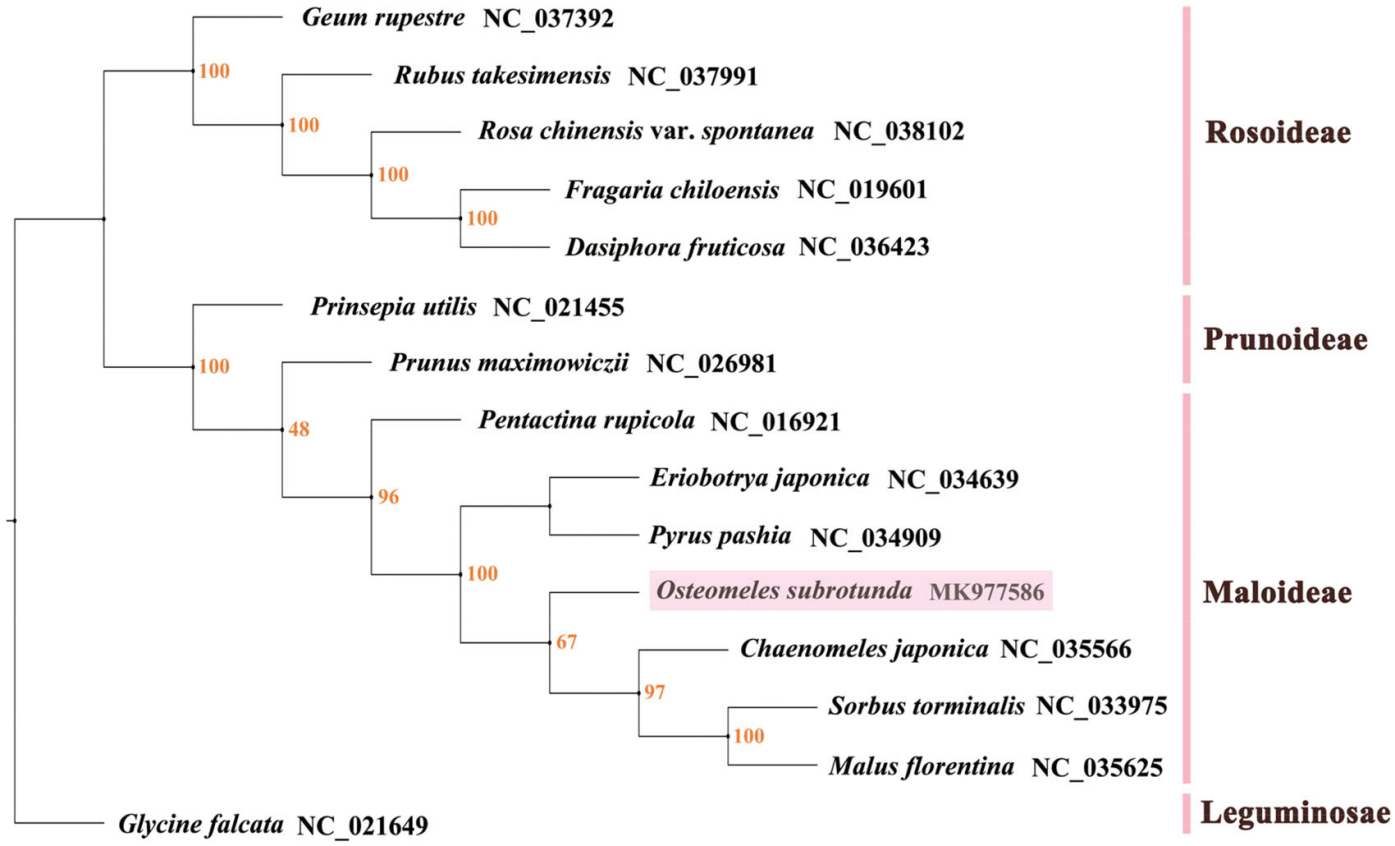
The maximum-likelihood tree inferred from 15 Rosaceae complete chloroplast genomes under GTR + G+I model with maximum-likelihood value (Llk)=–578919.18882, Akaike information criterion (AIC)=1157904.93474, and Bayesian information criterion (BIC)=1158279.20184.

jModelTest was used to carry out the statistical selection of the best model of nucleotide substitution, and the results determined that the best-fit model was GTR + G+I. To determine the phylogenetic position of *O. subrotunda*, we constructed a phylogenetic tree using the whole CP genomes of 14 species in the family Rosaceae using *G. falcata* as an outgroup (Figure 3). Seven nodes were completely supported by 100% bootstrap, and two nodes had more than 95% bootstrap values. The phylogenic tree showed that the 14 Rosaceae species were divided into three groups, in which *G. rupestre*, *R*. *takesimensis*, *R. chinensis* var. *spontanea*, *F. chiloensis*, and *D. fruticose* in subfamily Rosoideae comprised one group, and *P. utilis* and *P. maximowiczii* in subfamily Prunoideae clustered another group. *O. subrotunda* grouped with *P. rupicola*, *E. japonica*, *P. pashia*, *C. japonica*, *S. torminalis*, and *M. florentina*, species belonging to subfamily Maloideae, and *O. subrotunda* was found to be a sister clade to *C. japonica* with a relative low bootstrap value of 67.

## Discussion

Based on our previous long-term investigation, *O. subrotunda* is a wild plant species with tiny populations. To protect this rare species, it was listed as a key protected plant in Zhejiang Province. According to our knowledge, there was no report on this plant species. The rapid development of modern sequencing platforms in recent years has significantly facilitated to CP genome research. Currently, more than 2000 CP genomes were deposited in GenBank (https://www.ncbi.nlm.nih.gov/genome/browse#!/organelles/). However, there are only 45 Rosaceae CP genomes in GenBank, which mainly include *Prunus* (13), *Fragaria* (10), *Malus* (6), and *Rosa* (6), while *Osteomeles* is not on the list. In this study, we sequenced and assembled the CP genome of *O. subrotunda*. Typically, CP genomes in angiosperms contain a conserved quadripartite circular structure, including two copies of IRs which were separated by an LSC and an SSC (Jansen et al. 2005; Du et al. 2017). Our results indicated that the complete CP genome of *O. subrotunda* was 159,902 bp in length, which contained a typical quadripartite structure. The full length of *O. subrotunda* is within the range of the CP genome of other Rosaceae plants (Jansen et al. 2011; Wang et al. 2013; Bao et al. 2016). Similar to known CP genome in Rosaceae, *O. subrotunda* contained more AT bases, which was smaller than that of *R. chinensis* var. *spontanea* but larger than *Sorbus torminalis* (Jian, Zhang, Yan, et al. 2018).

SSR markers are tandemly repeated nucleotide sequence consisting copies of mono-, di-, tri-, or tetranucleotide motifs flanked by unique sequences (McCouch et al. 1997). SSR sequences distribute in nuclear genomes, and they are widely used in genetic diversity analysis, linkage mapping studies, and marker-assisted breeding (Kaur et al. 2015; Almontero and Espino 2016; Tian et al. 2017; Ahmad et al. 2018; Chao et al. 2018). Likewise, CP genome sequences have been found to contain SSRs, and they have been increasingly used in both population genetic structure and evolutionary studies for their high polymorphism (Kikuchi et al. 2013; Wheeler et al. 2014; Fu et al. 2016; Takahashi et al. 2018). *Musa balbisiana* CP genome possesses 59 SSRs, though mono-nucleotides are common (29 SSRs, accounts for 49.15% of the total SSRs), it also includes di-, tri-, tetra-, penta-, and hexa-nucleotides (Shetty et al. 2016). In strawberry CP genome, 61 SSR loci are detected, among which 38 are mono-nucleotide repeats, 16 are di-repeats, besides, there are three tri-repeats and four tetra-repeats (Cheng et al. 2017). While the CP genome of *O. subrotunda*, a total of 61 SSRs were identified; however, there were only two types of nucleotide repeats, mono- and di-nucleotide repeats, respectively.

Generally, gene content, gene order, and genome structure in CP genomes are highly conserved in flowering plants; however, gene loss, pseudogenization, and IR expansion or contraction are also common during CP genome evolution (Wicke et al. 2011; Daniell et al. 2016). Gene duplication in plastids of some hemiparasite plants, *ndhC* is found missing in both *Striga hermonthica* and *S. aspera*, while ndhF is missing only in *Buchnera americana* (Frailey et al. 2018). In the three Actinidiaceae plants, *Actinidia polygama*, *A. tetramera*, and *Clematoclethra lanosa*, their *clpP* genes were absent in CP genomes (Wang et al. 2016). However, in our present study, gene loss was not observed. The expansion and contraction of IRs account for CP genome size variations and gene pseudogenization (Ni et al. 2016; Wang et al. 2016). The IRs underwent both expansion and contraction during evolution in Rosaceae family, among which *D. fruticosa* CP genome showed the shortest IR region (25,311 bp), while *P. maximowiczii* demonstrated the longest IR sequence (26,436 bp). Pseudogenization commonly occur in parasitic, semi-parasitic, and non-parasitic plant species (Raman and Park 2015; Daniell et al. 2016; Gruzdev et al. 2016; Shin and Lee 2018). In *Dendrotrophe varians* CP genome, *infA* was found to be a pseudogene which contained a premature stop codon, moreover, at the LSC-IRA junction, the *rps19* was truncated at its 3′ end (Shin and Lee 2018). The *ndhH* gene of *Dendrocalamus latiflorus* crossed IRa/SSC boundaries, creating an incomplete copy of *ndhH* gene (Wu et al. 2015). In our present study, *ycf1* located at IRB/SSC border was a pseudogene with its 3′ region truncated, and the other *ycf1* gene of *C. japonica* at LSC/IRA border was also proved to be a pseudogene.

## Data Availability

The data that support the findings of this study are openly available in GenBank of NCBI at https://www.ncbi.nlm.nih.gov/nuccore/MK977586. The associated BioProject, SRA, and Bio-Sample numbers are PRJNA685556, SRR13275017, and SAMN17087660, respectively.

## References

[CIT0001] Ahmad A, Wang JD, Pan YB, Sharif R, Gao SJ. 2018. Development and use of simple sequence repeats (SSRs) markers for sugarcane breeding and genetic studies. Agronomy. 8(11):260.

[CIT0002] Allen JF. 2015. Why chloroplasts and mitochondria retain their own genomes and genetic systems: colocation for redox regulation of gene expression. Proc Natl Acad Sci USA. 112(33):10231–10238. 26286985 10.1073/pnas.1500012112 PMC4547249

[CIT0003] Almontero CC, Espino RRC. 2016. Genetic fingerprinting of onion (*Allium cepa* L.) varieties using simple sequence repeat markers. Philipp J Crop Sci. 41:22–32.

[CIT0004] Bao L, Li K, Liu Z, Han M, Zhang D. 2016. Characterization of the complete chloroplast genome of the Chinese crabapple *Malus prunifolia* (Rosales: Rosaceae: Maloideae). Conserv Genet Resour. 8(3):227–229.

[CIT0005] Chao WZ, Tang CH, Zhang JS, Yu L, Yoichi H. 2018. Development of a stable SCAR marker for rapid identification of *Ganoderma lucidum* Hunong 5 cultivar using DNA pooling method and inter-simple sequence repeat markers. J Integr Agric. 17(1):130–138.

[CIT0006] Cheng H, Li J, Zhang H, Cai B, Gao Z, Qiao Y, Mi L. 2017. The complete chloroplast genome sequence of strawberry (*Fragaria*×*ananassa* Duch.) and comparison with related species of Rosaceae. PeerJ. 5:e3919. 29038765 10.7717/peerj.3919 PMC5641433

[CIT0007] Daniell H, Lin CS, Yu M, Chang WJ. 2016. Chloroplast genomes: diversity, evolution, and applications in genetic engineering. Genome Biol. 17(1):134. 27339192 10.1186/s13059-016-1004-2 PMC4918201

[CIT0008] Darriba D, Taboada GL, Doallo R, Posada D. 2012. jModelTest 2: more models, new heuristics and parallel computing. Nat Methods. 9(8):772. 10.1038/nmeth.2109 PMC4594756 22847109

[CIT0009] Dierckxsens N, Mardulyn P, Smits G. 2017. NOVOPlasty: de novo assembly of organelle genomes from whole genome data. Nucleic Acids Res. 45(4):e18. 28204566 10.1093/nar/gkw955 PMC5389512

[CIT0010] Dong W, Xu C, Cheng T, Zhou S. 2013. Complete chloroplast genome of *Sedum sarmentosum* and chloroplast genome evolution in Saxifragales. PLOS One. 8(10):e77965. 24205047 10.1371/journal.pone.0077965 PMC3799696

[CIT0011] Dorrell RG, Howe CJ. 2012. What makes a chloroplast? Reconstructing the establishment of photosynthetic symbioses. J Cell Sci. 125(Pt 8):1865–1875. 22547565 10.1242/jcs.102285

[CIT0012] Doyle JJ, Doyle JL. 1987. A rapid DNA isolation procedure for small quantities of fresh leaf tissue. Phytochem Bull. 19:11–15.

[CIT0013] Du YP, Bi Y, Yang FP, Zhang MF, Chen XQ, Xue J, Zhang XH. 2017. Complete chloroplast genome sequences of *Lilium*: insights into evolutionary dynamics and phylogenetic analyses. Sci Rep. 7(1):5751. 28720853 10.1038/s41598-017-06210-2 PMC5515919

[CIT0014] Frailey DC, Chaluvadi SR, Vaughn JN, Coatney CG, Bennetzen JL. 2018. Gene loss and genome rearrangement in the plastids of five hemiparasites in the family Orobanchaceae. BMC Plant Biol. 18(1):30. 29409454 10.1186/s12870-018-1249-x PMC5801802

[CIT0015] Fu J, Liu H, Hu J, Liang Y, Liang J, Wuyun T, Tan X. 2016. Five complete chloroplast genome sequences from *Diospyros*: genome organization and comparative analysis. PLOS One. 11(7):e0159566. 27442423 10.1371/journal.pone.0159566 PMC4956199

[CIT0016] Gichira AW, Li Z, Saina JK, Long Z, Hu G, Gituru RW, Wang Q, Chen J. 2017. The complete chloroplast genome sequence of an endemic monotypic genus *Hagenia* (Rosaceae): structural comparative analysis, gene content and microsatellite detection. PeerJ. 5:e2846. 28097059 10.7717/peerj.2846 PMC5228516

[CIT0017] Gruzdev EV, Mardanov AV, Beletsky AV, Kochieva EZ, Ravin NV, Skryabin KG. 2016. The complete chloroplast genome of parasitic flowering plant *Monotropa hypopitys*: extensive gene losses and size reduction. Mitochondrial DNA B Resour. 1(1):212–213. 33473455 10.1080/23802359.2016.1155090 PMC7800667

[CIT0018] Guindon S, Dufayard JF, Lefort V, Anisimova M, Hordijk W, Gascuel O. 2010. New algorithms and methods to estimate maximum-likelihood phylogenies: assessing the performance of PhyML 3.0. Syst Biol. 59(3):307–321. 20525638 10.1093/sysbio/syq010

[CIT0019] Hsieh CF, Chaw SM. 1996. Osteomeles schwerinae C. K. Schneid. (Rosaceae): a new record for the flora of Taiwan. Bot Bull Acad Sin. 37:281–285.

[CIT0020] Jansen RK, Raubeson LA, Boore JL, dePamphilis CW, Chumley TW, Haberle RC, Wyman SK, Alverson AJ, Peery R, Herman SJ, et al. 2005. Methods for obtaining and analyzing whole chloroplast genome sequences. Methods Enzymol. 395:348–384. 15865976 10.1016/S0076-6879(05)95020-9

[CIT0021] Jansen RK, Saski C, Lee SB, Hansen AK, Daniell H. 2011. Complete plastid genome sequences of three rosids (Castanea, Prunus, Theobroma): evidence for at least two independent transfers of rpl22 to the nucleus. Mol Biol Evol. 28(1):835–847. 20935065 10.1093/molbev/msq261 PMC3108605

[CIT0022] Jian HY, Zhang SD, Zhang T, Qiu XQ, Yan HJ, Li SB, Wang QG, Tang KX. 2018. Characterization of the complete chloroplast genome of a critically Endangered decaploid rose species, *Rosa praelucens* (Rosaceae). Conserv Genet Resour. 10(4):851–854.

[CIT0023] Jian HY, Zhang YH, Yan HJ, Qiu XQ, Wang QG, Li SB, Zhang SD. 2018. The complete chloroplast genome of a key ancestor of modern roses, *Rosa chinensis* var. spontanea, and a comparison with congeneric species. Molecules. 23(2):389. 10.3390/molecules23020389 PMC6017658 29439505

[CIT0024] Jung S, Main D. 2014. Genomics and bioinformatics resources for translational science in Rosaceae. Plant Biotechnol Rep. 8(2):49–64. 24634697 10.1007/s11816-013-0282-3 PMC3951882

[CIT0025] Katoh K, Standley DM. 2013. MAFFT multiple sequence alignment software version 7: improvements in performance and usability. Mol Biol Evol. 30(4):772–780. 23329690 10.1093/molbev/mst010 PMC3603318

[CIT0026] Kaur S, Panesar PS, Bera MB, Kaur V. 2015. Simple sequence repeat markers in genetic divergence and marker-assisted selection of rice cultivars: a review. Crit Rev Food Sci Nutr. 55(1):41–49. 24915404 10.1080/10408398.2011.646363

[CIT0027] Kikuchi R, Pak JH, Takahashi H, Maki M. 2013. Pattern of population genetic structure revealed by nuclear simple sequence repeat markers in the understory perennial *Veratrum album* ssp. *oxysepalum* (Melanthiaceae) with a disjunct pattern of chloroplast DNA haplotypes. Biol J Linn Soc Lond. 108(2):278–293.

[CIT0028] Lee J, Jang DS, Yoo NH, Lee YM, Kim JH, Kim JS. 2010. Single-step separation of bioactive flavonol glucosides from *Osteomeles schwerinae* by high-speed counter-current chromatography. J Sep Sci. 33(4–5):582–586. 20127917 10.1002/jssc.200900693

[CIT0029] Lohse M, Drechsel O, Kahlau S, Bock R. 2013. OrganellarGenomeDRAW—a suite of tools for generating physical maps of plastid and mitochondrial genomes and visualizing expression data sets. Nucleic Acids Res. 41(Web Server Issue):W575–W581. 23609545 10.1093/nar/gkt289 PMC3692101

[CIT0030] Martin G, Baurens FC, Cardi C, Aury JM, D'Hont A. 2013. The complete chloroplast genome of banana (*Musa acuminata*, Zingiberales): insight into plastid monocotyledon evolution. PLOS One. 8(6):e67350. 23840670 10.1371/journal.pone.0067350 PMC3696114

[CIT0031] McCouch SR, Chen X, Panaud O, Temnykh S, Xu Y, Cho YG, Huang N, Ishii T, Blair M. 1997. Microsatellite marker development, mapping and applications in rice genetics and breeding. Plant Mol Biol. 35(1–2):89–99. 9291963

[CIT0032] Ni L, Zhao Z, Xu H, Chen S, Dorje G. 2016. The complete chloroplast genome of *Gentiana straminea* (Gentianaceae), an endemic species to the Sino-Himalayan subregion. Gene. 577(2):281–288. 26680100 10.1016/j.gene.2015.12.005

[CIT0033] Patel RK, Jain M. 2012. NGS QC Toolkit: a toolkit for quality control of next generation sequencing data. PLOS One. 7(2):e30619. 22312429 10.1371/journal.pone.0030619 PMC3270013

[CIT0034] Raman G, Park S. 2015. Analysis of the complete chloroplast genome of a medicinal plant, *Dianthus superbus* var. *longicalyncinus*, from a comparative genomics perspective. PLOS One. 10(10):e0141329. 26513163 10.1371/journal.pone.0141329 PMC4626046

[CIT0035] Shetty SM, Shah MUM, Makale K, Mohd-Yusuf Y, Khalid N, Othman RY. 2016. Complete chloroplast genome sequence of *Musa balbisiana* corroborates structural heterogeneity of inverted repeats in wild progenitors of cultivated bananas and plantains. Plant Genome. 9(2):1–14. 10.3835/plantgenome2015.09.0089 27898825

[CIT0036] Shi L, Chen H, Jiang M, Wang L, Wu X, Huang L, Liu C. 2019. CPGAVAS2, an integrated plastome sequence annotator and analyzer. Nucleic Acids Res. 47(W1):W65–W73. 31066451 10.1093/nar/gkz345 PMC6602467

[CIT0037] Shi LX, Theg SM. 2013. The chloroplast protein import system: from algae to trees. Biochim Biophys Acta. 1833(2):314–331. 23063942 10.1016/j.bbamcr.2012.10.002

[CIT0038] Shin HW, Lee NS. 2018. Understanding plastome evolution in hemiparasitic santalales: complete chloroplast genomes of three species, *Dendrotrophe varians*, *Helixanthera parasitica*, and *Macrosolen cochinchinensis*. PLOS One. 13(7):e0200293. 29975758 10.1371/journal.pone.0200293 PMC6033455

[CIT0039] Sohn E, Kim J, Kim CS, Jo K, Kim JS. 2018. *Osteomeles schwerinae* extract prevents diabetes-induced renal injury in spontaneously diabetic torii rats. Evid Based Complement Alternat Med. 2018:6824215. 29849722 10.1155/2018/6824215 PMC5941800

[CIT0040] Takahashi D, Sakaguchi S, Isagi Y, Setoguchi H. 2018. Comparative chloroplast genomics of series Sakawanum in genus *Asarum* (Aristolochiaceae) to develop single nucleotide polymorphisms (SNPs) and simple sequence repeat (SSR) markers. J Forest Res. 23(6):387–392.

[CIT0041] Thiel T, Michalek W, Varshney RK, Graner A. 2003. Exploiting EST databases for the development and characterization of gene-derived SSR-markers in barley (*Hordeum vulgare* L.). Theor Appl Genet. 106(3):411–422. 12589540 10.1007/s00122-002-1031-0

[CIT0042] Tian HY, Yan JQ, Channa SA, Zhang RJ, Guo Y, Hu SW. 2017. Analysis of the A genome genetic diversity among *Brassica napus*, *B. rapa* and *B. juncea* accessions using specific simple sequence repeat markers. Pak J Bot. 49:125–132.

[CIT0043] Wang S, Shi C, Gao LZ. 2013. Plastid genome sequence of a wild woody oil species, *Prinsepia utilis*, provides insights into evolutionary and mutational patterns of Rosaceae chloroplast genomes. PLOS One. 8(9):e73946. 24023915 10.1371/journal.pone.0073946 PMC3759469

[CIT0044] Wang WC, Chen SY, Zhang XZ. 2016. Chloroplast genome evolution in Actinidiaceae: clpP loss, heterogenous divergence and phylogenomic practice. PLOS One. 11(9):e0162324. 27589600 10.1371/journal.pone.0162324 PMC5010200

[CIT0045] Waters MT, Langdale JA. 2009. The making of a chloroplast. EMBO J. 28(19):2861–2873. 19745808 10.1038/emboj.2009.264 PMC2744177

[CIT0046] Wheeler GL, Dorman HE, Buchanan A, Challagundla L, Wallace LE. 2014. A review of the prevalence, utility, and caveats of using chloroplast simple sequence repeats for studies of plant biology. Appl Plant Sci. 2(12):1400059. 10.3732/apps.1400059 PMC4259455 25506520

[CIT0047] Whittall JB, Syring J, Parks M, Buenrostro J, Dick C, Liston A, Cronn R. 2010. Finding a (pine) needle in a haystack: chloroplast genome sequence divergence in rare and widespread pines. Mol Ecol. 19:100–114. 20331774 10.1111/j.1365-294X.2009.04474.x

[CIT0048] Wicke S, Schneeweiss GM, dePamphilis CW, Müller KF, Quandt D. 2011. The evolution of the plastid chromosome in land plants: gene content, gene order, gene function. Plant Mol Biol. 76(3–5):273–297. 21424877 10.1007/s11103-011-9762-4 PMC3104136

[CIT0049] Wu ML, Lan SR, Cai BP, Chen SP, Chen H, Zhou SL. 2015. The complete chloroplast genome of *Guadua angustifolia* and comparative analyses of neotropical-paleotropical bamboos. PLoS One. 10(12):e0143792. 26630488 10.1371/journal.pone.0143792 PMC4668023

[CIT0050] Wu ZY, Raven PH, Hong DY. 2003. Flora of China. Vol. 9. Beijing and St. Louis: Science Press and Missouri Botanical Garden Press.

